# Abscisic acid at the crossroad of abiotic stress responses and plant-microbe interactions

**DOI:** 10.1371/journal.ppat.1013872

**Published:** 2026-01-23

**Authors:** Athanasios Makris, Beril Daloglu, Caroline Gutjahr, Martina K. Ried-Lasi

**Affiliations:** 1 Department of Molecular Signal Processing, Leibniz Institute of Plant Biochemistry, Symbiosis Signalling Group, Halle (Saale), Germany; 2 Department of Root Biology and Symbiosis, Max Planck Institute of Molecular Plant Physiology, Potsdam, Germany; University of Tübingen: Eberhard Karls Universitat Tubingen, GERMANY

## ABA signalling in a changing environment

Plants are constantly exposed to complex combinations of abiotic and biotic stresses that threaten their growth, survival, and productivity [1]. Enhancing plant resistance, tolerance, and resilience to withstand and recover from environmental perturbations, is essential for maintaining agricultural productivity and ecological stability, especially in an era of global change.

The key hormone abscisic acid (ABA) plays a central role in plant responses to abiotic stressors such as drought, salinity, and cold [2]. Traditionally recognised for its involvement in leaf and fruit abscission, dehydration responses, and seed dormancy, ABA is now understood as a dynamic regulator of broader stress physiology. In addition to its well-established roles in abiotic stress adaptation, emerging evidence reveals ABA also modulates plant immunity and influences interactions with both pathogenic and beneficial microbes [3–5]. Through intricate crosstalk with other hormone signalling pathways, including salicylic acid (SA) and jasmonic acid (JA), ABA coordinates growth-defence trade-offs and integrates multiple environmental cues [6,7].

Understanding how ABA orchestrates these diverse responses is key to unravelling the molecular basis of plant adaptation. In this *Pearls* article, we highlight insights into ABA’s dual role in stress physiology and plant-microbe interactions and examine how this hormone fine-tunes plant resilience in a changing environment.

## ABA as a central regulator of abiotic stress tolerance in plants

Plants encounter a wide range of abiotic stresses throughout their life cycles, including drought, salinity, and cold. These often occur simultaneously, compounding their effects on metabolism, growth, and reproduction [8]. ABA is a central player in enabling plant adaptation to such conditions by activating both immediate physiological responses and long-term transcriptional reprogramming.

ABA biosynthesis is tightly regulated in response to environmental cues [9]. The first step of ABA biosynthesis involves the production of zeaxanthin by ß-carotene hydroxylation. Next, zeaxanthin epoxidase (ZEP) is required for the conversion of zeaxanthin to violaxanthin. After neoxanthin is synthesised from violaxanthin, nine-*cis*-epoxycarotenoid dioxygenase (NCED) enzymes are required for the cleavage of the *cis*-isomers of these products into xanthoxin, the precursor of abscisic aldehyde that is oxidised into ABA [10]. Under drought stress, the *ZEP* gene is upregulated in roots of Arabidopsis, tomato and *Nicotiana plumbaginifolia,* while some *NCED* genes respond in both roots and leaves of *Phaseolus vulgaris*, Arabidopsis, tomato, and *N. plumbaginifolia* [11–13]. Moreover, the *LOS5/ABA3* gene encodes a molybdenum cofactor sulfurase that activates aldehyde oxidase (AO), the enzyme catalysing the final oxidation step of ABA biosynthesis. In *los5/aba3* mutants, AO remains inactive, resulting in reduced ABA accumulation under drought stress. Since ABA is required to induce many osmotic stress-responsive genes, the reduced ABA levels in *los5/aba3* mutants lead to attenuated activation of ABA-dependent genes. Finally, ABA is essential for triggering stomatal closure through PYR/PYL-PP2C-SnRK2 and ABA mutants fail to efficiently activate this pathway leading to impaired drought-induced stomatal closure and higher transpirational water loss [14].

**Fig 1:**
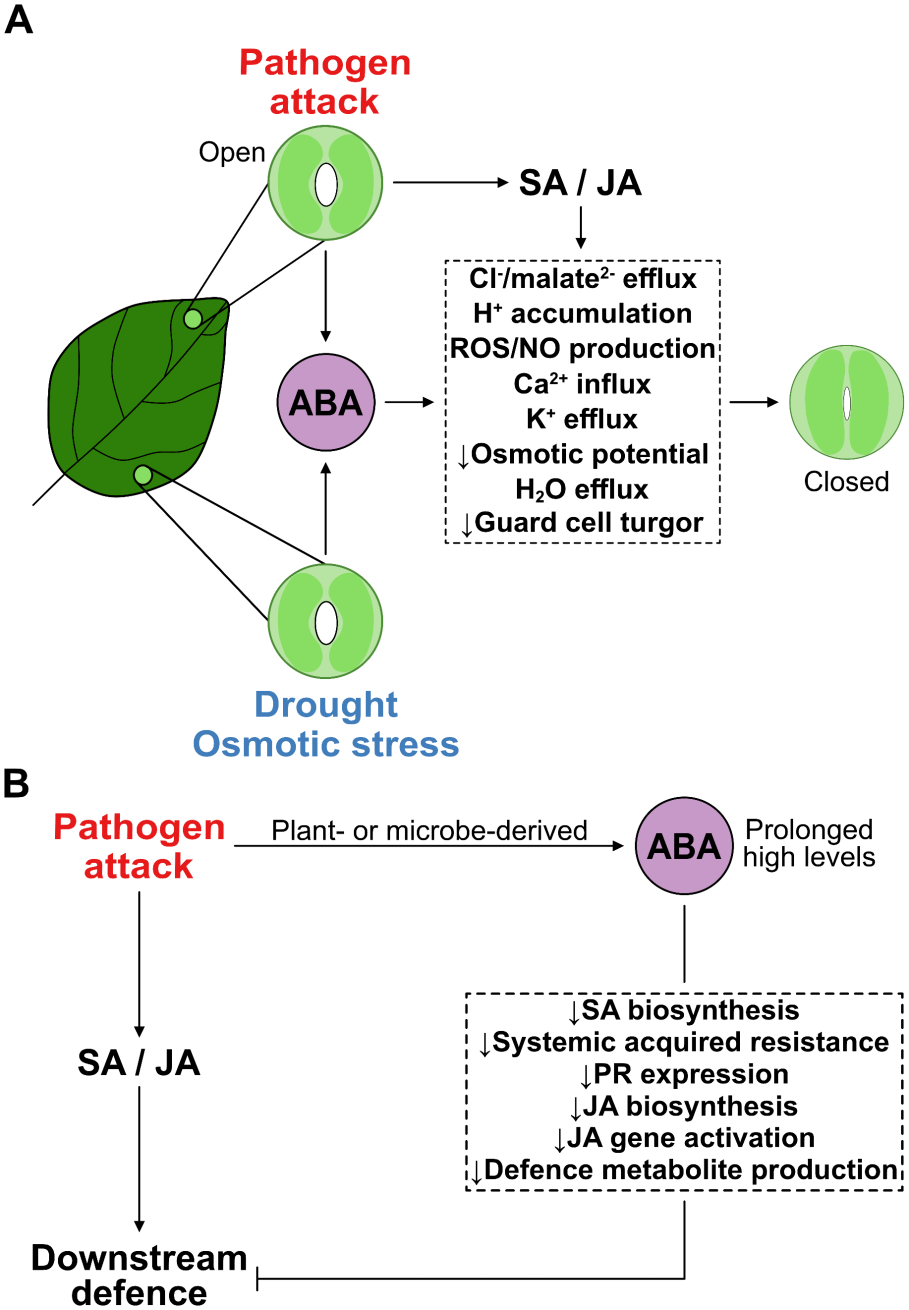
Intersections in ABA functions in abiotic stress response and pathogen defence. **(A)** ABA accumulates in drought or osmotic stress conditions and in the early phase of some pathogen attacks and promotes stomatal closure. It activates RCAR/PYL receptors after binding, which activate SnRK2. SnRK2 triggers a cascade of metabolite and ion fluxes into and out of stomatal guard cells as well as production of ROS/NO, which act as second messengers at multiple levels of stomatal closure regulation [25,26]. In more detail, SnRK2 phosphorylates and thereby activates Ca^2+^ efflux channels in the vacuolar membrane leading to Ca^2+^ influx into the cytoplasm. Ca^2+^ activates malate channels and Cl^−^ channels in the plasma membrane, the latter of which are additionally activated by phosphorylation through SnRK2. In addition, plasma membrane ATPases are inhibited by Ca^2+^ leading to accumulation of protons in the cytoplasm and its acidification. Together the malate²^−^ and Cl^−^ accumulation in the guard cell apoplast as well as the proton accumulation in the guard cell cytoplasm lead to membrane depolarisation, causing K^+^ efflux channels to open. The activated massive malate²^−^, Cl^−^, and K^+^ efflux from the cell leads to a drop in osmotic potential of the guard cells and an increase of osmotic potential of their surrounding apoplast. This osmotic imbalance between inside and outside of guard cells causes the efflux of water, loss of guard cell turgor and stomatal closure. Thereby transpiration is reduced. SA and JA are plant hormones with prominent roles in pathogen defence. Upon pathogen attack the levels of SA and/or JA increase. By binding to their receptors (NPR1 and COI1 respectively) SA and JA trigger the same metabolite and ion fluxes as ABA to induce stomatal closure and to reduce pathogen entry via stomata. **(B)** Besides its role in promoting stomatal closure and increasing pre-invasion defence, ABA can also negatively interfere with pathogen defence [27,28]. ABA can suppress SA and JA biosynthesis, leading to reduction in systemic acquired resistance, PR gene expression and defence metabolite accumulation and thereby a reduction in defence.

ABA is perceived by RCAR/PYR/PYL proteins, which upon ABA-binding interact with and inhibit the activity of PROTEIN PHOSPHATASES TYPE 2C (PP2Cs) proteins, which in absence of ABA inhibit SUCROSE NON-FERMENTING 1-RELATED PROTEIN KINASE2 (SnRK2) through their phosphatase activity. Overexpression of RCAR ABA receptors leads to increased water use efficiency without growth penalty in Arabidopsis and wheat [15,16]. When upon ABA-binding by RCAR/PYR/PYL the SnRK2 kinases are liberated from inhibition by PP2Cs, they activate ion channels for rapid responses such as stomatal closure (Fig 1A) and/or they activate transcriptional responses in the nucleus [17]. ABA (or SnrK2)-responsive transcription factors such as MYC/MYB, ABF2/AREB1, DREB2A/DREB2B, RD22BP1, and ZF-HD bind to *cis*-regulatory elements in target genes and activate stress-responsive pathways that promote cellular homeostasis [10,18]. Beyond cellular regulation, ABA drives morphological adaptations to abiotic stress including changes in root-system architecture [19,20], and improves hydraulic conductivity, aiding in water acquisition from deeper soil layers [21–24].

## ABA in biotic stress: barrier defence and immune modulation

ABA also plays complex and context-dependent roles in plant responses to biotic stress. One clearly beneficial contribution is its involvement in pre-invasion defence, especially through promoting stomatal closure (Fig 1A). This response helps block foliar pathogens like the hemibiotrophic bacterium *Pseudomonas syringae* from entering leaf tissue [29]. ABA also contributes to cuticle reinforcement, increasing the thickness and integrity of the epidermal barrier and reducing the success of pathogens that rely on physical penetration [30].

However, ABA can also antagonise immune signalling pathways, particularly those involving SA and JA, two key regulators of pathogen defence (Fig 1B) [27,28]. For instance, ABA suppresses SA biosynthesis by downregulating the transcription factors CBP60g and SARD1, which are essential for SA production and immune activation including systemic acquired resistance [31,32]. Notably, CBP60g and SARD1 are active during early and late stages of bacterial infection [33], highlighting ABA’s potential to dampen immune responses across infection phases. Moreover, the expression of PATHOGENESIS-RELATED proteins, which are part of the plant’s inducible immune response and the production of which is strongly increased when a plant is attacked by a pathogen, as well as flg22-triggered callose formation, both hallmarks of SA signalling, are suppressed by endogenous as well as exogenous ABA (Fig 1B) [34]. Flg22 is derived from the N‑terminal region of a bacterial protein called flagellin, which is the main building block of bacterial flagella.

ABA’s impact on plant immunity is influenced by pathogen lifestyle. While it often increases susceptibility to biotrophic pathogens, which rely on living host tissue and are typically countered by SA-mediated defences, it may enhance defence against necrotrophic pathogens (e.g., *Pythium irregulare*, *Alternaria brassicicola*) that kill host tissue to feed on the resulting cellular debris, by cooperating with the JA signalling pathway [35].

Yet, ABA can also favour susceptibility to necrotrophs like *Botrytis cinerea* [36] likely by suppressing JA-responses (Fig 1B), especially through interactions with JAZ repressors. Molecularly, the ABA receptor PYL6 physically interacts with MYC2, a major transcriptional regulator of JA-responses, altering downstream signalling dynamics. This interaction promotes the expression of JAZ8, a transcriptional repressor of JA-responses, and decreases the activity of JAZ6, thereby selectively modulating the intensity and nature of JA signalling (Fig 1B) [37]. Similarly, it has been shown recently that ABA can increase the expression of another JAZ repressor, JAZ1, which binds to NAC42-type transcriptional activators consequently suppressing the production of phytoalexins that act as defence metabolites (Fig 1B) [38].

Intriguingly, several pathogens exploit ABA signalling, including synthesising and sensing ABA via specific receptors, in order to facilitate colonisation or evade immune detection [39–41] (Fig 1B). *P. syringae* pv. tomato induces ABA accumulation to suppress SA-mediated defences and promote infection [42]. Similarly, *Magnaporthe oryzae* enhances ABA biosynthesis in host tissues and may produce ABA itself, suggesting a pathogen-derived virulence strategy based on ABA [43]. Moreover, ABA has been shown to enhance fungal chitin deacetylase activity in submerged cultures of *Colletotrichum gloeosporioides* [44]. Fungal chitin deacetylases catalyse the deacetylation of chitin resulting in the formation of chitosan [45–47], which has been described as a major virulence strategy especially in soil borne fungal pathogens [48,49].

ABA signalling can also be exploited by the plant immune system without actual ABA perception. In effector triggered immunity, nucleotide binding leucine rich repeat receptors (NLRs) bind pathogen effectors and are in many cases triggered to induce cell death [50]. The tomato NLR Sw-5b (belonging to the CC-NB-LRR family), recognises the effector NSm of *Tomato spotted wilt orthotopovirus*. Interestingly, effector perception triggers direct binding of Sw-5b to protein phosphatase 2C4 (PP2C4). This inhibits PP2C4 such that SnRK2 is released from inhibition and ABA responses promoting defense against viral infections are induced. Thus, Sw-5b acts as ABA receptor mimic in the context of Tomato *spotted wilt orthotopovirus* infection [51].

In summary, ABA’s impact on biotic stress is multifaceted, beneficial in some contexts, immunosuppressive in others, depending on the pathogen, timing, tissue, and environmental background. As such, ABA functions as both a guardian of pre-invasion barriers and a modulator of downstream immune signalling, making it a central, but double-edged, player in plant-pathogen interactions (Fig 1).

## ABA as a regulator of beneficial plant-microbe interactions

Plant interactions with beneficial microorganisms are vital for nutrient acquisition, stress tolerance, and overall health. They include microbial symbionts such as nitrogen-fixing bacteria (e.g., of the genus Rhizobium) and arbuscular mycorrhiza fungi (AMF, of the clade Glomeromycotina). AMF form widespread mutualistic relationships with over 80% of terrestrial plant species called arbuscular mycorrhiza (AM), while rhizobia engage in root nodule symbiosis with legumes. The symbioses improve plant nutrition and can be beneficial to plants under abiotic stress and for defence against pathogens [52–54].

**Fig 2:**
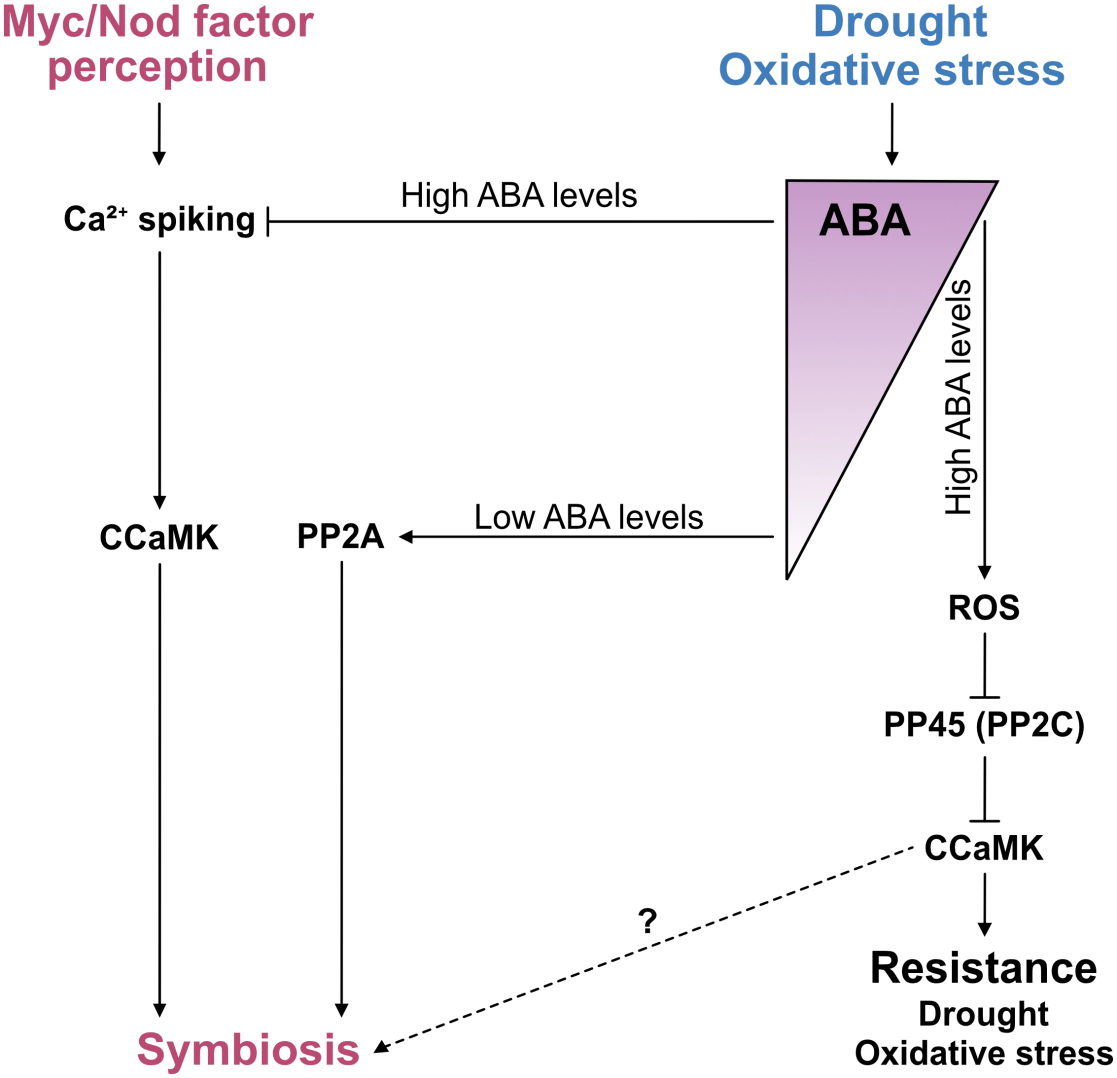
ABA affects root symbiosis in a dose dependent manner and promotes CCaMK activity in leaves. Low ABA levels promote arbuscular mycorrhiza (AM) symbiosis via protein phosphatase 2A (PP2A) through an unknown mechanism [56]. High ABA levels in turn suppress AM as well as root nodule symbiosis by inhibiting nuclear calcium oscillations (calcium spiking) that are triggered after perception of Myc- and Nod-Factors (chitin-oligomer based signals from AM fungi and rhizobia, respectively) by the plant cell [56,57]. The mechanism for the inhibition of calcium spiking by ABA is unknown. Calcium spiking is believed to be deciphered by Calcium and Calmodulin-dependent Kinase (CCaMK), a central positive regulator of both AM and root nodule symbiosis [59]. CCaMK also plays a role in ABA-mediated abiotic stress responses in leaves of rice [60,61]. Upon drought and oxidative stress, ABA signalling leads to an increase in reactive oxygen species (ROS) production which inhibits the protein phosphatase 2C (PP2C) called PP45. This releases CCaMK from inhibition by dephosphorylation such that CCaMK can promote resistance to drought and osmotic stress. It remains to be studied whether ABA also promotes CCaMK activity in the context of root symbiosis.

It has been suggested that ABA promotes AM symbiosis and more precisely arbuscule formation and viability in tomato [55]. In *Medicago truncatula,* low ABA levels enhance AM colonisation, requiring the regulatory subunit B’1 of the Protein Phosphatase 2A (PP2A) holoenzyme (Fig 2) [56]. Mutants lacking this subunit show reduced AM colonisation despite displaying normal symbiotic calcium spiking in response to Myc factors, (lipo)chito-oligosaccharides that are produced by AMF [56]. Conversely, high levels of ABA interfere with symbiotic calcium spiking and impair AM development [56], revealing a dose dependent regulatory mechanism paralleling the effect of high concentrations of ABA inhibiting calcium spiking triggered by bacterial Nod-factors in the context of nitrogen-fixing root nodule symbiosis (Fig 2) [57]. In apple roots it was observed that ABA promotes AM by enhancing lipid biosynthesis required to feed the fungus via the ABA-responsive transcription factor ABF2 [58].

Calcium and calmodulin-dependent kinase (CCaMK) is a central component of common symbiosis signalling [62], which is shared between AM and nitrogen-fixing root nodule symbiosis and is required for root colonisation by both rhizobia and AM fungi [63]. CCaMK is thought to act as a decoder of symbiotic calcium spiking triggered by Myc or Nod-factors [59]. Interestingly in rice leaves, CCaMK appears to be involved in mediating ABA responses thereby regulating drought stress and oxidative stress adaptation and also seed germination and root growth independently of root symbioses (Fig 2) [61,64]. In this context, ABA activates CCaMK through release from inhibitory dephosphorylation by the PP2C-type phosphatase PP45, which in turn is inhibited by oxidation through ABA-induced H_2_O_2_ (Fig 2) [60]. Then CCaMK phosphorylates OsMKK1 and activates the MAPK cascade [61]. This suggests a mechanism that acts in parallel with Ca^2+^ and calmodulin and by which ABA during abiotic stress increases CCaMK activity by preventing CCaMK dephosphorylation. It will be interesting to understand whether ABA also promotes symbiosis via CCaMK at least at low concentrations that do not inhibit calcium spiking.

Interestingly, in bean plants, root colonisation by the symbiotic endophyte *Metarhizium robertsii* reduced ABA and its metabolites, increased stomatal size, and repressed the expression of immunity genes, while infection with the pathogenic fungus *Fusarium solani* had the opposite effects [65]. In addition, exogenous application of ABA further decreased *M. robertsii* colonisation but promoted *F. solani* colonisation [65]. Taken together, this indicates a central role of ABA in mediating contrasting responses to symbiotic versus pathogenic fungi.

## Conclusions

Abscisic acid sits at the nexus of abiotic and biotic stress signalling. It mediates fundamental responses to dehydration, salinity, and oxidative stress, while also influencing the outcome of pathogen encounters and symbiotic relationships with microbes. Through hormonal crosstalk, transcriptional regulation, and redox signalling, ABA fine-tunes stress responses and resource allocation.

ABA signalling cross-talks with other hormonal pathways to balance growth, defence, symbiosis, and survival. Its effects are context- and ABA level-dependent, shaped by pathogen lifestyle, stress combinations, and tissue-specific dynamics.

Decoding the full scope of ABA’s role in this crosstalk remains a key challenge. As climate change increases the frequency and complexity of stress combinations, understanding how ABA integrates a diversity of inputs will be essential for developing resilient crops and sustainable agricultural systems.
